# Pronator quadratus–sparing volar plating of distal radius fractures: A prospective study

**DOI:** 10.1051/sicotj/2026036

**Published:** 2026-10-08

**Authors:** Ahmed Nouh, Mohammad H. Amer, Hassan Magdy El-Barbary, Ahmed Al-Feeshawy, Mohamed Abdel-Wahed

**Affiliations:** 1 Orthopaedic Department, Helwan University Cairo Egypt; 2 Orthopaedic Department, Faculty of Medicine, Cairo University Cairo Egypt

**Keywords:** Distal radius fracture, Minimally invasive surgery, Pronator quadratus, Volar locking plate, Wrist fractures

## Abstract

*Purpose:* Volar locked plates for the distal radius fractures (DRFs) are applied through the Henry approach, and its modification entails a routine step to disinsert the pronator quadratus from its radial and distal attachments. This muscle insertion is mostly fleshy with minimal tendinous tissue, and surgeons find it difficult to reattach at the end of the surgery, the hardware comes in direct contact with the flexor tendons. *Methods:* The study was performed as a single-center prospective case series to investigate the possibility of preserving the pronator quadratus and, at the same time, achieving good reduction. Out of 73 patients enrolled in the study, the pronator quadratus was successfully spared in 60 patients. The quality of reduction and fixation in these patients was assessed functionally and radiologically. *Results:* Cases showed radiographic union commencing 3 months postoperatively. Twenty-nine cases had to wait for the 6th months follow up radiographs. No case had a nonunion of its fracture. The PQ sparing approach can be shifted to the FCR approach when needed. In our series, the pronator quadratus muscle was taken down in 4 patients, where a satisfactory reduction could not be achieved. *Conclusion:* PQ sparing approach is readily adopted and can be used for the majority of DRFs, achieving a good functional and radiological outcome. Its use can enhance recovery and reduce hardware-related complications. Nonetheless, reduction should not be compromised, and the PQ sparing can be converted readily to the FCR approach.

## Introduction

Distal radius fractures (DRFs) account for about 17.5% of all adult fractures and represent the most common fracture of the upper extremity [1]. For severely displaced or unstable fractures, open reduction and internal fixation (ORIF) is often indicated [2]. Volar locking plate fixation through the flexor carpi radialis (FCR) approach is widely used and considered a standard technique for distal radius fixation [3]. Classically, the pronator quadratus (PQ) muscle is released from the radial side of the distal radius to expose the fracture and is later repaired after plate fixation [4]. However, this repair is often not feasible or reliable [4]. Some authors have suggested that failure to restore the PQ may leave the plate exposed to the flexor tendons, increasing the risk of tendon irritation or rupture [5, 6].

To address these concerns, minimally invasive PQ-sparing approaches have been developed. Imatani et al. [7] first described a technique for volar plating without detaching the PQ, and Dos Remedios et al. [8] reported a similar approach. Theoretically, preserving the PQ creates a cushion between the plate and the flexor tendons and maintains the muscle’s function. Several studies have reported better early functional outcomes and less pain post-operatively with PQ-sparing than the traditional approach [9, 10].

In this prospective case series at a Level I trauma center with a dedicated hand surgery service, we evaluated the PQ-sparing volar plating technique for distal radius fractures. Our objectives were to determine whether the PQ can be preserved without compromising fracture reduction, to describe how a volar locking plate can be applied without releasing the PQ, to identify potential benefits of PQ preservation, and to assess any drawbacks of this approach.

## Patients and methods

### Study design and patients

This study was a single-center prospective case series conducted after institutional review board approval. We enrolled skeletally mature adults (age ≥ 18 years) with isolated, closed distal radius fractures who were neurovascularly intact and elected operative treatment. Patients were excluded for fractures >3 weeks old, age >60 years (reflecting our practice of treating most low-demand elderly patients nonoperatively unless the fracture was highly unstable), or pathologic fractures (e.g., metastatic lesions). Notably, the status of the PQ muscle was not an exclusion criterion; cases with a contused but intact PQ were still attempted with the PQ-sparing technique. All patients provided written informed consent.

Operative fixation was indicated for any of the following: an articular step-off >2 mm, volar displacement, an initial dorsal displacement with high instability risk (dorsal tilt >10°, metaphyseal comminution, or ulnar variance >3 mm), or early loss of reduction after closed management. All fractures were classified according to the 2018 AO/OTA system. We included AO/OTA types 23-A, 23-B, and selected 23-C fractures (specifically 23-C1 and C2). Highly comminuted fractures (e.g., AO 23-C3) that typically require fragment-specific fixation were excluded preoperatively and managed with the standard PQ-releasing approach.

### Surgical technique

All procedures were performed under regional anesthesia with the patient in the supine position and the affected limb placed on a radiolucent hand table. A pneumatic tourniquet was applied after administration of a first-generation cephalosporin.

A standard flexor carpi radialis (FCR) approach was used. A longitudinal volar incision of approximately 6 cm was made over the FCR tendon. After opening the sheath, the tendon was retracted ulnarly, and the flexor pollicis longus was gently mobilized to expose the pronator quadratus (PQ). The PQ was released only from its distal radial attachment, preserving the remaining muscle fibers (Figures 1A, 1B).

**Figure 1:**
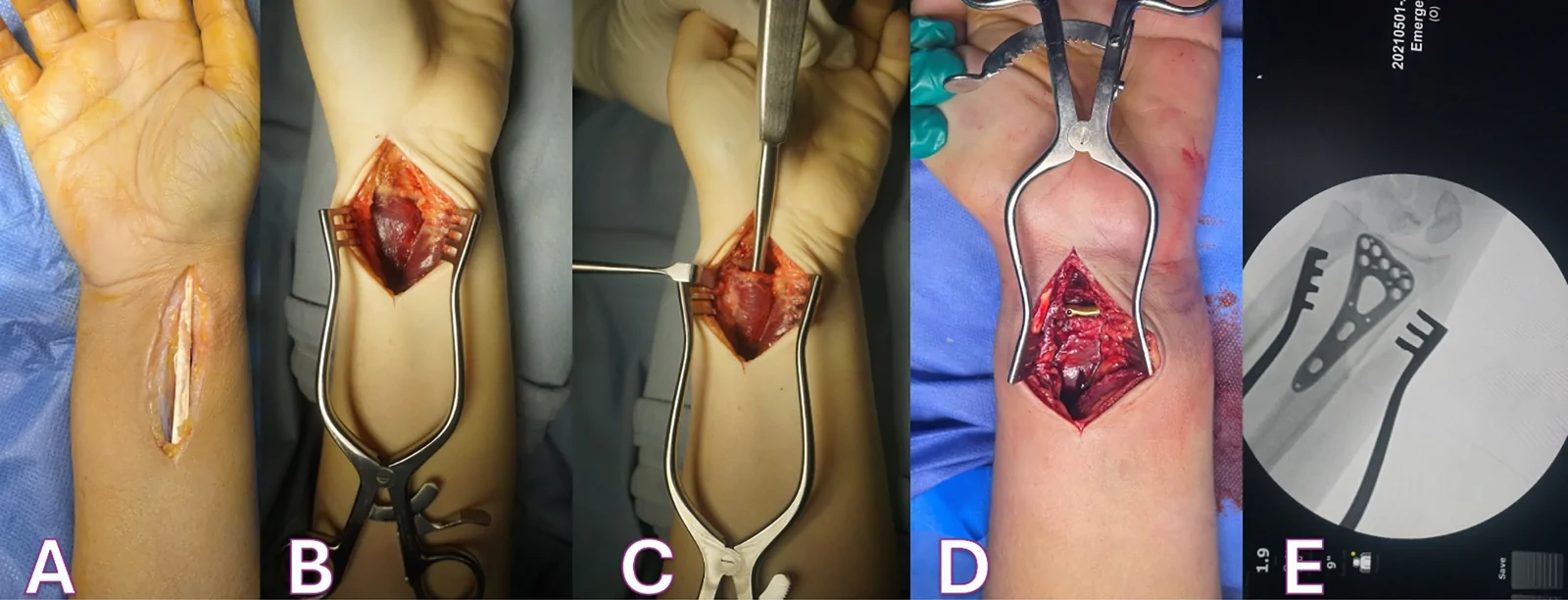
Intraoperative photographs and fluoroscopic images demonstrating the PQ-sparing surgical technique. (A) Exposure of the FCR tendon. (B) The FCR tendon is retracted to expose the PQ. (C) Incising the distal tip of the PQ and creating a tunnel under it. (D) Sliding the plate retrograde under the tunnel created. (E) Fluoroscopic views showing the placement of the volar locking plate under the intact PQ.

A polyaxial anatomic volar locking plate was inserted retrogradely beneath the intact PQ and positioned on the volar aspect of the distal radius under fluoroscopic guidance (Figures 1C–1E). The plate was temporarily fixed to the distal fragment using a Kirschner wire. Fracture reduction was achieved using traction and ligamentotaxis, with coronal alignment confirmed on the anteroposterior fluoroscopic view.

To restore sagittal alignment, a small transverse split was made in the PQ fibers over the elongated proximal plate slot. A non-locking cortical screw was inserted through this slot into the radial shaft. Tightening of this screw secured the plate to the bone and facilitated restoration of volar tilt, which was confirmed on the lateral fluoroscopic view. Additional proximal locking screws were then inserted through small stab incisions in the PQ. Distal locking screws were placed into the elevated distal fragment through the area where the PQ had already been released, without the need for further muscle dissection.

Final anteroposterior and lateral fluoroscopic images confirmed satisfactory fracture reduction and appropriate plate positioning. The tourniquet was released, meticulous hemostasis was achieved, and the wound was closed in layers.

In cases of severe comminution, indirect reduction techniques were employed, including manual traction, joystick Kirschner wires, and use of the plate as a reduction template, while preserving the periosteum and intact PQ as stabilizing structures. If an acceptable reduction could not be achieved with the PQ preserved – such as persistent malalignment or fracture gapping on fluoroscopy – the approach was converted to the conventional technique with complete release of the PQ to allow direct visualization and reduction.

### Postoperative care

A removable wrist splint was used for comfort for the first 2 weeks post-op. Patients began active finger, elbow, and shoulder motion immediately, and gentle wrist motion as tolerated. They were followed up at 2, 4, and 6 weeks. By 6 weeks, fracture stability allowed most patients to resume daily activities as tolerated (avoiding sports/strenuous use until ~12 weeks). We recorded patient and therapist feedback on pain and function during early recovery, including analgesic consumption (from medication logs).

For long-term evaluation, at 1 year post-op, we assessed the Disability of Arm, Shoulder, and Hand (DASH) score, grip strength (using a dynamometer), and wrist range of motion (ROM) with a goniometer. The contralateral uninjured wrist served as a control for ROM and strength, with results expressed as a percentage of the opposite side. Radiological outcomes were evaluated on final follow-up radiographs by two independent observers, measuring standard parameters (volar tilt, radial inclination, and ulnar variance).

Some patients were followed for a minimum of 1 year (up to 4 years in a few cases, especially those who requested hardware removal). Notably, plate removal was also performed with a PQ-sparing approach. In those cases, the PQ was found to be well-healed and covering the plate, with a substantial portion of its muscle belly intact, confirming that the muscle had remained viable and protective over the hardware (Figure 2).

**Figure 2:**
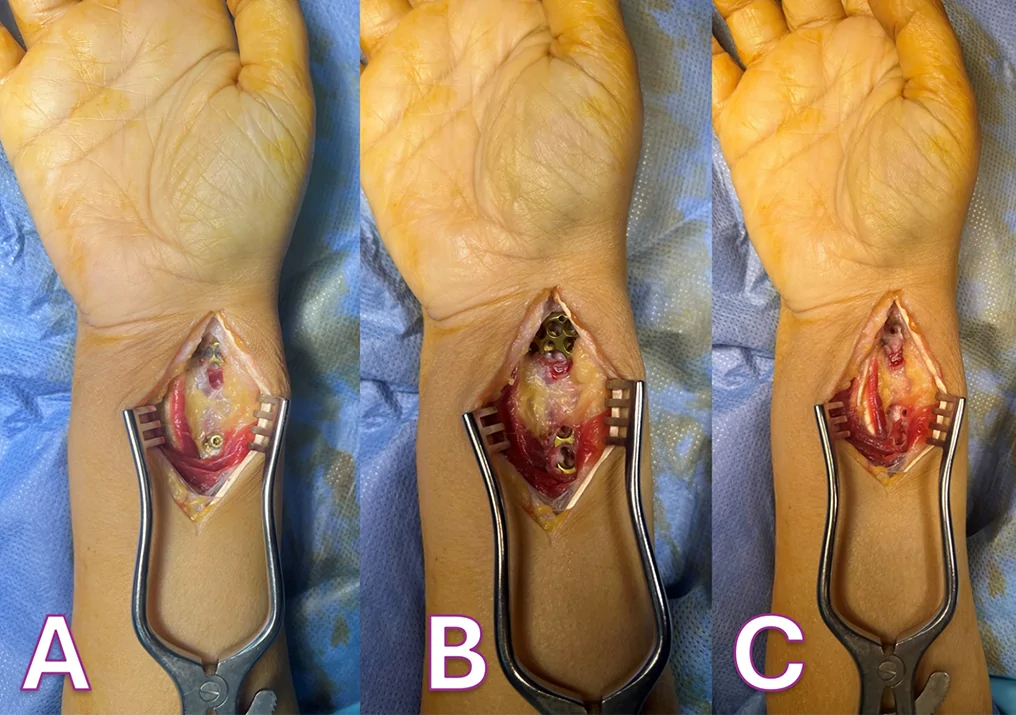
(A) Photograph of a patient undergoing plate removal with a PQ-sparing approach, showing the well-healed PQ muscle covering the plate. (B) The screws are removed through small incisions over the PQ. (C) The plate is removed while the PQ is spared.

### Statistical analysis

We used descriptive statistics to summarize the data. Continuous variables are reported as means with ranges (or medians with ranges as noted). No formal hypothesis testing was performed, given the single-cohort design. All analyses were done using SPSS version 25 (IBM Corp., Armonk, NY).

## Results

From March 2018 to November 2024, 81 patients met the initial inclusion criteria. Eight of these were managed with the standard PQ-releasing approach and were excluded from analysis (two required fragment-specific fixation for very complex patterns, and six had severely comminuted fractures deemed unsuitable for PQ-sparing). The remaining 73 patients were treated with the intent to spare the PQ. In three patients, the PQ was already ruptured by the initial injury (despite appearing intact preoperatively) and could not be preserved. In six cases, the PQ partially tore intraoperatively during manipulation or plate insertion. In four patients, the surgeon elected to release the PQ because an adequate reduction could not be achieved with the muscle intact (e.g., a persistent dorsal tilt or gap on fluoroscopy). Thus, of 73 fractures in which the PQ-sparing technique was attempted, the PQ was successfully preserved in 60 (82%) (Figure 3).

**Figure 3:**
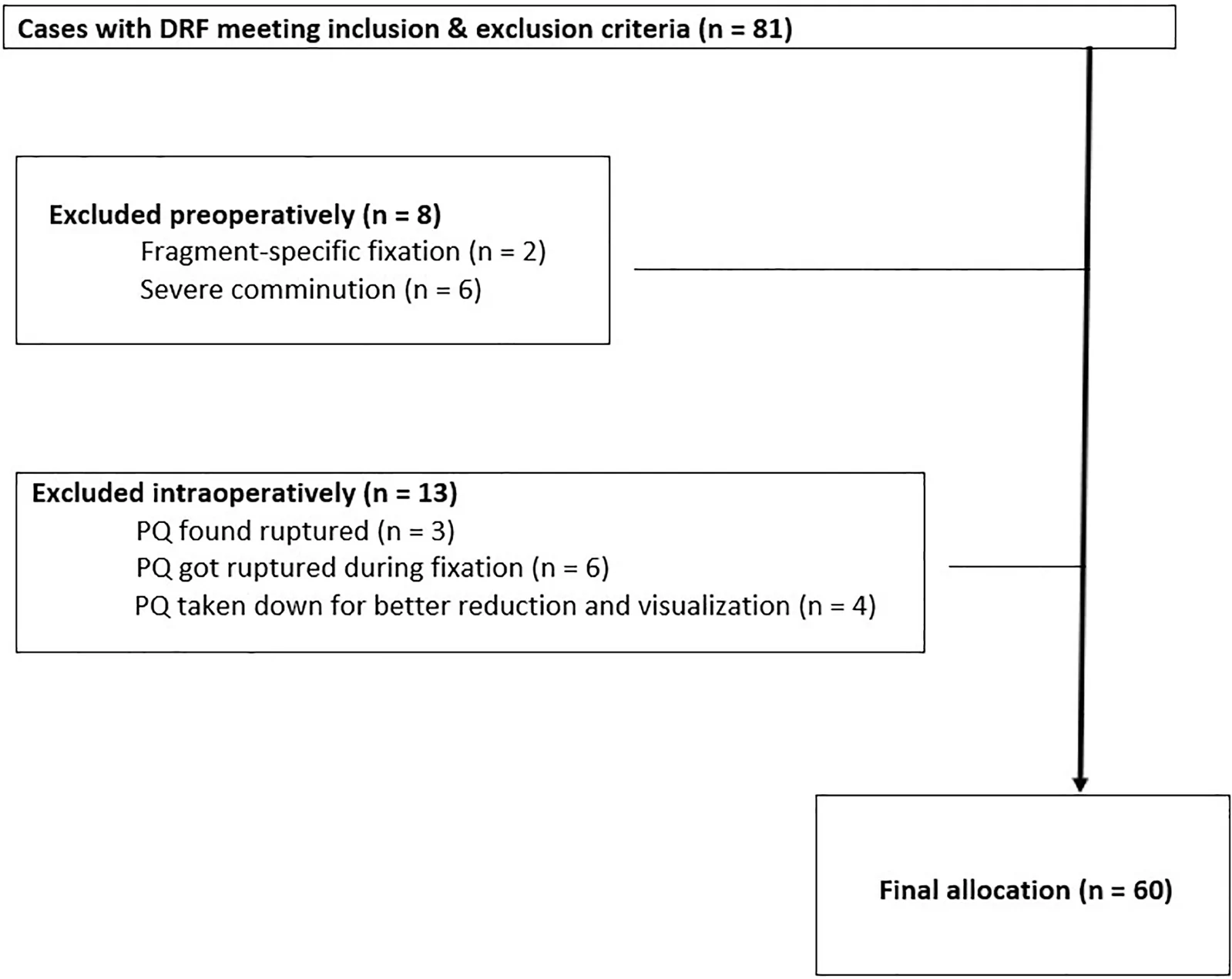
Flowchart illustrating patient enrollment and reasons for exclusion, detailing the number of patients in whom the PQ-sparing technique was successfully used.

The mean age of the 73 patients was 38 years (range 21–55). The cohort included 48 men and 25 women; in the PQ-preserved subgroup (*n* = 60), there were 36 men and 24 women. Sixty-five percent of fractures were AO/OTA type 23-B, 22% type 23-A, and 13% type 23-C. All eight type C fractures in the PQ-sparing group were simple (C1 or C2); complex C3 fractures were among those excluded. High-energy mechanisms (motor vehicle accidents or falls from height) accounted for 65% of injuries, and low-energy falls for 35%. Mean follow-up was 18 months (range 12–45). The mean time from injury to surgery was 3.2 days (range 1–7). The mean operative time (skin incision to closure) was 68 min (range 45–100) (Table 1).

**Table 1:** Patient demographics and surgical data.

Variable	Value
Age, years (mean ± SD, range)	38 ± 8 (21–55)
Sex, *n* (%)	Male: 36 (60%); Female: 24 (40%)
Fracture classification (AO/OTA), *n* (%)	23-A: 13 (22%); 23-B: 39 (65%); 23-C1/C2: 8 (13%)
Mechanism of injury, *n* (%)	High-energy: 39 (65%); Low-energy: 21 (35%)
Time from injury to surgery, days (mean ± SD, range)	3.2 ± 1.7 (1–7)
Operative time, minutes (mean ± SD, range)	68 ± 15 (45–100)
Follow-up duration, months (mean ± SD, range)	18 ± 8 (12–45)

All fractures healed radiographically. By 2 months postoperatively, 44 patients showed early union, and all remaining fractures were united by 4 months. No patient developed nonunion. At 12-month follow-up, the mean volar tilt was 6° volar (range 0° dorsal to 12° volar), the mean radial inclination was 22° (range 16°–25°), and the mean ulnar variance was +0.5 mm (range −1 to +2 mm). By standard criteria, none of the fractures met the definition of malunion (dorsal tilt >10°, ulnar variance >3 mm, or radial inclination <15°). In other words, PQ preservation did not prevent us from obtaining or maintaining an acceptable alignment (Table 2).

**Table 2:** Radiographic and functional outcomes at 12 months.

Outcome measure	12-month value
Volar tilt, degrees (mean ± SD, range)	6 ± 4 (0° dorsal to 12° volar)
Radial inclination, degrees (mean ± SD, range)	22 ± 2 (16–25)
Ulnar variance, mm (mean ± SD, range)	+0.5 ± 1.0 (−1 to +2)
Flexion–extension arc (% of contralateral, mean ± SD, range)	91 ± 5 (84–100)
Pronation–supination arc (% of contralateral, mean ± SD, range)	99 ± 2 (96–100)
Grip strength (% of contralateral, mean ± SD, range)	81 ± 12 (50–100)
DASH score at 6 months (median, range)	7.5 (0–50)
DASH score at 12 months (median, range)	3 (0–20)

Functional recovery was excellent. The median DASH score was 7.5 at 6 months and 3 at 12 months, indicating near-normal function. At final follow-up, nine patients (15%) had regained full wrist range of motion (flexion–extension and pronation–supination) compared to the uninjured side. The remaining patients had minimal deficits: the mean flexion–extension arc was 91% of the contralateral side (range 84–100%), and forearm rotation was essentially symmetric (mean pronation 98% and supination 99% of the opposite side, with no patient losing >10° in either plane). Mean grip strength at 1 year was 81% of the uninjured wrist. No patient reported any noticeable loss of forearm pronation strength or endurance.

Complications were infrequent. Two patients developed postoperative hematomas, both of which resolved with conservative management, and one patient had a superficial wound infection that resolved with oral antibiotics. One patient developed complex regional pain syndrome, which improved with therapy and medication. Notably, there were no cases of hardware impingement, flexor tendon irritation or rupture, or median nerve neuropathy in this series (Table 3).

**Table 3:** Postoperative complications.

Complication	*n* (%)
Postoperative hematoma	2 (3.3%)
Superficial wound infection	1 (1.7%)
Complex regional pain syndrome (CRPS)	1 (1.7%)
Tendon irritation or rupture	0 (0%)
Median nerve neuropathy	0 (0%)

## Discussion

Our experience confirms that a PQ-sparing volar plating approach is technically feasible in most distal radius fractures, but it is not appropriate for every case. The surgeon’s priority must always be to achieve an anatomic reduction and stable fixation, and this should not be compromised solely to preserve the PQ. In our practice, we preoperatively identified about 10% of fractures as unsuitable for PQ-sparing (for example, very comminuted patterns requiring fragment-specific fixation). Even with careful patient selection, 4% of patients had a PQ muscle already torn from the injury, and another 8% had an intraoperative PQ tear, reflecting reports that PQ tears occur in up to ~12% of distal radius fractures [11]. These cases illustrate that some injuries inherently preclude PQ preservation.

Importantly, we elected to convert to the standard approach in four patients because the reduction was inadequate with the PQ intact. In each of those cases, releasing the PQ allowed completion of an anatomic reduction. Ultimately, all fractures in our series united with acceptable alignment (no malunions). This is consistent with prior reports showing no difference in final radiographic parameters between PQ-sparing and conventional approaches [9], and even one study reporting slightly better restoration of volar tilt with PQ preservation [12]. It has been hypothesized that an intact PQ (and periosteum) can act as an internal splint or provide resistance to maintain reduction. Our intraoperative experience supports this idea: with the PQ preserved, it provided gentle resistance to displacement of fragments and, in some cases, appeared to aid indirect reduction in multifragmentary fractures.

We observed excellent functional outcomes, with low DASH scores and nearly full motion by 1 year. Comparable results have been reported elsewhere [9, 13]. For example, some series have found that PQ-sparing patients recover wrist motion and function faster in the early postoperative period than those treated with the conventional approach, although differences tend to disappear after a few months [9]. In one randomized trial and a recent meta-analysis, PQ-sparing (or PQ repair) was associated with less early pain and slightly better function in the first 2–3 months, but by 6–12 months, there was no significant difference in function or range of motion [9, 14]. These findings suggest that preserving the PQ mainly reduces immediate postoperative pain and speeds early recovery, benefits that are certainly valuable, even though by about 8–12 weeks, functional measures such as ROM, strength, and DASH scores are similar regardless of PQ handling [13]. In our series (which lacked a control group), we saw rapid early recovery and excellent 1-year results, aligning with this concept. We did not observe any patient with significant forearm pronation weakness. This is noteworthy because one prior study reported a modest decrease in pronation strength at 6 months when the PQ was detached [15]. However, another meta-analysis found no consistent long-term difference in pronation strength between patients with and without PQ repair. We did not systematically measure pronation strength, but no patient complained of weakness.

A potential advantage of the PQ-sparing approach is reduced risk of flexor tendon complications. Volar plate prominence beyond the watershed line, residual malalignment, and absence of a PQ barrier have all been identified as risk factors for flexor tendon irritation or rupture [16]. Failure to repair or maintain the PQ has been reported as an even stronger risk factor for tendon injury than plate position in one series [17]. In our cohort, with the PQ preserved in most cases (and loosely draped over the plate even when partially released), we had no flexor tendon ruptures or notable tendon irritation. This is consistent with other reports; for example, one comparative study found 0% flexor irritation in a PQ-sparing group versus about 7.6% in a PQ-released group (though the difference was not statistically significant) [13]. These findings suggest that maintaining a muscular layer over the plate can confer a protective effect for the flexor tendons.

Several alternative minimally invasive techniques to spare the PQ have been described. Kashir and O’Donnell introduced a brachioradialis-splitting approach, which elevates the PQ from the radial side while splitting the brachioradialis, allowing plate insertion without detaching the PQ [18]. Zhang et al. compared the standard FCR-based PQ-sparing approach to the brachioradialis-splitting approach and found no significant differences in outcomes, although the brachioradialis approach had a slightly higher incidence of transient superficial radial nerve symptoms [19]. These techniques point to the growing interest in minimizing soft-tissue trauma during distal radius fixation.

This study has limitations. The most important limitation is the absence of a direct control group treated with the conventional PQ-releasing approach. As a single-cohort series, we cannot quantify the magnitude of benefit provided by PQ preservation compared to the standard technique. Another limitation is that our series included relatively few complex intra-articular (AO 23-C3) fractures, since such fractures were generally excluded or converted to standard fixation. Thus, our conclusions primarily apply to fracture types amenable to PQ-sparing (extra-articular and simple intra-articular patterns). On the other hand, with 60 patients in whom the PQ was successfully preserved, our series is among the largest reported for this technique to date. Future randomized trials or larger comparative studies will be valuable to determine definitively whether PQ preservation offers any additional long-term advantages or reduction in complication rates.

## Conclusion

A pronator quadratus–sparing volar plating approach can be used for most distal radius fractures, yielding excellent radiographic and functional outcomes. Preserving the PQ appears to enhance early recovery (by minimizing muscle trauma and postoperative pain) and may help protect flexor tendons by retaining a soft-tissue barrier over the plate. However, the quality of reduction must remain the top priority: if an anatomic reduction cannot be achieved with the PQ intact, the surgeon should not hesitate to release the PQ. In our experience, routine preservation of the PQ when feasible did not entail any disadvantages. We therefore recommend preserving the PQ muscle whenever possible during volar plating of distal radius fractures, provided it does not compromise the quality of reduction and fixation. Level of Evidence: IV (prospective case series).

## Data Availability

The data that support the findings of this study are available upon reasonable request from the corresponding author.
